# LiNi_0.5_Mn_1.5_O_4_ Cathode Microstructure for All-Solid-State Batteries

**DOI:** 10.1021/acs.nanolett.2c02426

**Published:** 2022-09-07

**Authors:** Hyeon Jeong Lee, Xiaoxiao Liu, Yvonne Chart, Peng Tang, Jin-Gyu Bae, Sudarshan Narayanan, Ji Hoon Lee, Richard J. Potter, Yongming Sun, Mauro Pasta

**Affiliations:** ‡ Department of Materials, University of Oxford, Oxford OX1 3PH, U.K.; ¶ The Faraday Institution, Harwell Campus, Quad One, Becquerel Avenue, Didcot OX11 0RA, United Kingdom; § Division of Chemical Engineering and Bioengineering, Kangwon National University, 1 Kangwondaehak-gil, Chuncheon 24341, Republic of Korea; ∥ Wuhan National Laboratory for Optoelectronics, Huazhong University of Science and Technology, Luoyu Road 1037, Wuhan, Hubei 430074, China; ⊥ School of Materials Science and Engineering, Kyungpook National University, Daegu 41566, Republic of Korea; # Department of Mechanical, Materials and Aerospace Engineering, University of Liverpool, Brownlow Street, Liverpool L69 3GH, United Kingdom

**Keywords:** cathode microstructure, solid-state batteries, areal capacities, high-voltage cathodes, interfaces

## Abstract

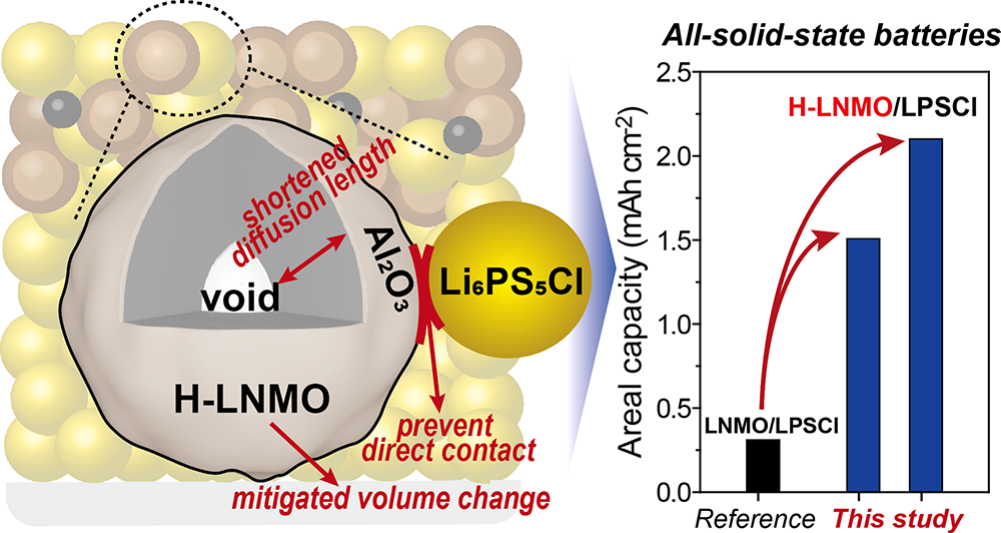

Solid-state batteries (SSBs) have received attention as a next-generation energy storage technology due to their potential to superior deliver energy density and safety compared to commercial Li-ion batteries. One of the main challenges limiting their practical implementation is the rapid capacity decay caused by the loss of contact between the cathode active material and the solid electrolyte upon cycling. Here, we use the promising high-voltage, low-cost LiNi_0.5_Mn_1.5_O_4_ (LNMO) as a model system to demonstrate the importance of the cathode microstructure in SSBs. We design Al_2_O_3_-coated LNMO particles with a hollow microstructure aimed at suppressing electrolyte decomposition, minimizing volume change during cycling, and shortening the Li diffusion pathway to achieve maximum cathode utilization. When cycled with a Li_6_PS_5_Cl solid electrolyte, we demonstrate a capacity retention above 70% after 100 cycles, with an active material loading of 27 mg cm^–2^ (2.2 mAh cm^–2^) at a current density of 0.8 mA cm^–2^.

Solid-state batteries (SSBs) are one of the most promising “beyond Li-ion” battery chemistries as they promise to fulfill the energy density, fast charging, and safety requirements of the future of electric transportation.^1,2^ The discovery of solid sulfide inorganic ceramic electrolytes in the early 2010s, with conductivities comparable to that of their liquid counterpart and mechanical properties amenable to scalable manufacturing, has further boosted the commercial interest in SSBs.^3−5^

Unfortunately, there are still several issues preventing SSBs from realizing their full potential.^6,7^ One of the most problematic challenges is the rapid capacity fade caused by the loss of contact between the Li-ion conductive solid electrolyte matrix and the active material in the composite cathode, which is triggered by volume changes that occur upon lithiation and delithiation.^8−10^ In addition, hoop stresses generated in the delithiated polycrystalline cathode as a result of the volume change of misoriented primary particles cause the formation of internal cracks.^11^ While these newly formed interfaces can be accessed by a liquid electrolyte, the limited plasticity of solid electrolytes prevents them from doing so, leading to continuous capacity decay.^12,13^ Low-strain cathodes have been reported to mitigate this problem by minimizing volume changes during cycling.^14−17^ Unfortunately, the existing low-strain cathode chemistries are incompatible with the energy density and cost requirements of commercial batteries. An alternative solution is represented by the microstructural design of high-energy cathode chemistries to mitigate the detrimental effects of volume expansion and achieve stable cycling.^18^

LiNi_0.5_Mn_1.5_O_4_ (LNMO) is one of the most promising cathode materials for next-generation lithium batteries due to its low cost (i.e., Co-free and Mn-rich) and high energy density (146 mAh g^–1^ theoretical capacity delivered at 4.7 V vs Li^+^/Li).^19,20^ The high operating potential of LNMO inevitably induces undesirable side reactions with a typical solid sulfide electrolyte, leading to the formation of highly resistive interphases.^21−23^ In addition, the volume change of LNMO during cycling is approximately 6.2%, which is higher than those of Ni-rich LiNi_*x*_Mn_*y*_Co_*z*_O_2_ (NMC) cathodes (5.1% for NMC811), thus making it an attractive model system for investigating the importance of microstructure design for SSBs.^24−27^

In this study, we introduce Al_2_O_3_-coated LNMO secondary particles designed to have a hollow microstructure and evaluate their electrochemical performance in an all-solid-state configuration (Figure 1a). Argyrodite Li_6_PS_5_Cl (LPSCl) was selected as the solid electrolyte because of its high ionic conductivity, inherent softness, and ability to form stable electrode–electrolyte interphases.^28−30^ A Li_0.25_-In_0.75_ (Li–In) alloy anode was used to mitigate interfacial issues commonly reported for metallic lithium and isolate the degradation mechanisms occurring at the cathode.^22,31^ The shortened Li-ion diffusion length of the hollow structure facilitates uniform Li-ion extraction and prevents internal stress from accumulating in the particle.^11,18,32^ The Al_2_O_3_ layer deposited by atomic layer deposition (ALD) effectively attenuates the interfacial side reaction with the solid LPSCl electrolyte, thus enabling stable cycling even at high operating potentials. The combination of the Al_2_O_3_ capping layer and the hollow microstructure alleviates the volume change of the LNMO particles, thus improving the long-term cyclability of the SSB. Cathode composites prepared by a scalable dry-milling process demonstrate capacity retention above 70% after 100 cycles, with an active material loading of 27 mg cm^–2^ (2.2 mAh cm^–2^) at a current density of 0.8 mA cm^–2^. To the best of our knowledge, this is the top-performing LNMO cathode in a SSB configuration.

**Figure 1:**
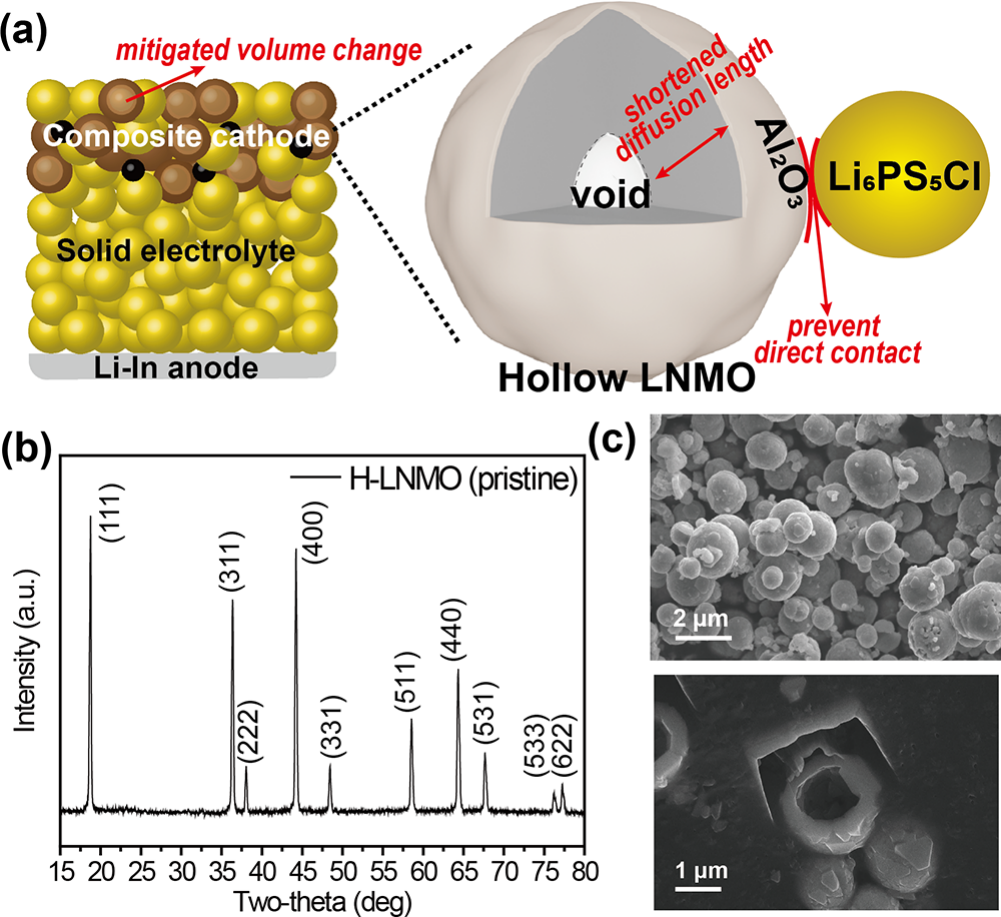
(a) Schematic illustration of the Al_2_O_3_-coated H-LNMO in the composite cathode of SSBs. (b) XRD pattern of H-LNMO. (c) SEM image of the synthesized H-LNMO particles and cross-section SEM image of the H-LNMO particles after FIB sectioning.

Hollow LNMO (H-LNMO) particles were synthesized via a two-step method previously reported by our group and described in detail in the Methods section of the Supporting Information.^18^ X-ray powder diffraction (XRD) of the as-synthesized H-LNMO particles confirms the synthesis of phase-pure LNMO with its characteristic cubic crystal structure (Figure 1b).^18,33,34^ Scanning electron microscopy (SEM) images show spherical secondary particles of H-LNMO that are a few micrometers in diameter and composed of plate-shaped primary particles with a size of about 500 nm (Figure 1c). SEM images of H-LNMO particles cross-sectioned by a focused ion beam (FIB) confirmed their hollow morphology and a shell thickness of approximately 400 nm (Figure 1c). Dynamic light scattering (DLS) analysis of the as-synthesized powder (Figure S1) showed D50 and D90 values of 3.62 and 5.29 μm, respectively, which are consistent with SEM observations.

Composite cathodes were prepared by mixing H-LNMO, LPSCl, and vapor-grown carbon fiber (VCF) in a weight ratio of 40:55:5, followed by a densification step at a uniaxial pressure of 500 MPa (see Methods section of the Supporting Information). Cross-section SEM images of the composite cathode show the intimate interfacial contact between H-LNMO and LPSCl and confirm that the hollow microstructure of LNMO was maintained after the densification step (Figure 2a). Energy dispersive X-ray spectroscopy (EDS) elemental mapping corroborates the homogeneous mixing of each component, which induces the formation of sufficient conduction pathways for both electrons and Li-ions (Figure 2b).

**Figure 2:**
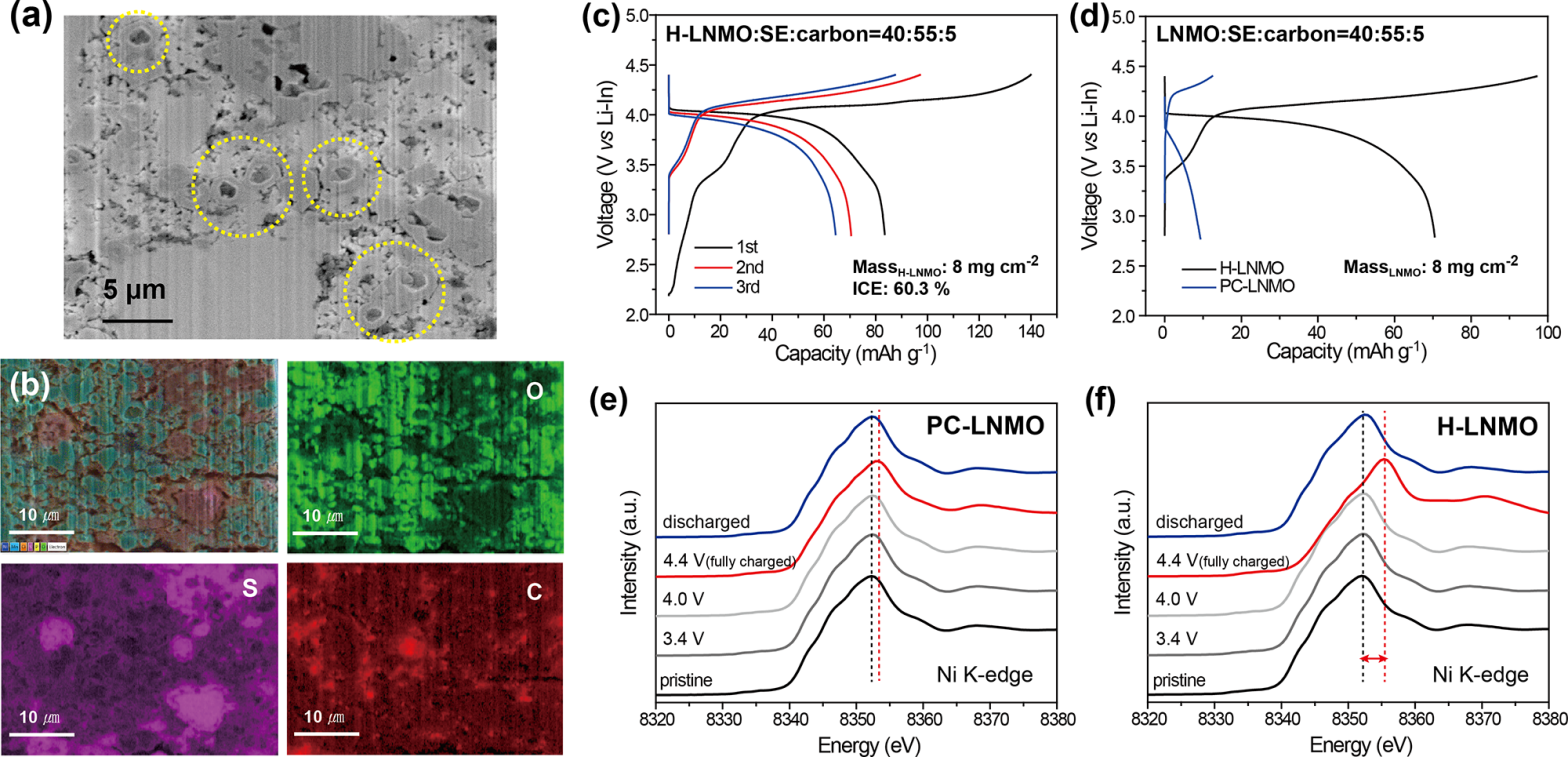
(a) Cross-section SEM image and (b) EDS elemental mappings of the H-LNMO/LPSCl/VCF (40:55:5) cathode composite. (c) GCD curves of SSBs with a H-LNMO composite (40:55:5) cycled at a rate of 0.1 C. (d) Comparative GCD curves of SSBs with H-LNMO and PC-LNMO composites at a rate of 0.1 C. Ex-situ XANES spectra measured at the Ni K edge of (e) PC-LNMO and (f) H-LNMO in pristine, 3.4, 4.0, 4.4 (fully charged), and 2.75 V (fully discharged) states.

The electrochemical performance of the H-LNMO composite cathode was tested in a two-electrode setup, where a Li–In alloy acted as both the reference and counter electrodes and a LPSCl pellet acted as the solid electrolyte separator (see the Methods section of the Supporting Information). Galvanostatic charge–discharge (GCD) curves of the H-LNMO composite cathode exhibit a first discharge capacity of 83.4 mAh g^–1^ with an initial Coulombic efficiency of 60.3%, as shown in Figure 2c. The two plateaus at 4.0 and 3.4 V (vs Li^+^/Li–In) correspond to the reduction of Ni^4+^ to Ni^2+^ and that of Mn^4+^ to Mn^3+^, respectively.^35,36^ The excess capacity on charge in the first cycle was attributed to LPSCl and VCF reacting at about 3.5 V to form a cathode–electrolyte interphase (CEI) composed of LiCl, S, and P_2_S_*x*_, which can act as a passivation layer in subsequent cycles.^37,38^ The GCD profile of the composite cathode without VCF confirms the absence of VCF-LPSCl side reactions (Figure S2).

In order to evaluate the effect of the hollow microstructure, the electrochemical properties of H-LNMO were compared to commercial polycrystalline LNMO (PC-LNMO). PC-LNMO secondary particles have a median particle size of 11 μm and are composed of primary particles with a size of 700 nm (Figure S3). They exhibited a discharge capacity of 10.1 mAh g^–1^ at a rate of 0.1 C and high voltage hysteresis (Figure 2d). We believe this behavior can be attributed to the longer diffusion length in PC-LNMO compared to that in the H-LNMO. This results in the faster formation of the Li-ion concentration gradient and consequently sets up an early trigger of the cutoff voltage. In addition, the larger particle size of PC-LNMO provides less contact area between the electrode particles and the solid electrolyte particles, which results in limited reaction kinetics and thus leads to a high overpotential and a reduced discharge capacity^12,39^ (Figure S4). Ex situ X-ray absorption near edge structure (XANES) analysis conducted on the pristine, charged, and discharged states of PC-LNMO and H-LNMO confirm the reversible redox activity of Ni (Figures 2e and f). However, the Ni redox swing in PC-LNMO is narrower than that in H-LNMO, thus confirming its partial (de)lithiation. To further clarify the effect of a shortened diffusion path in H-LNMO, single-crystalline LNMO (SC-LNMO, MTI) with a particle size of 3.8 μm was also electrochemically evaluated. SC-LNMO showed a discharge capacity of 21.1 mAh g^–1^ at a rate of 0.1 C, which was still far below the the discharge capacity of H-LNMO, again highlighting the effect of the hollow microstructure of H-LNMO (Figure S5).

To mitigate the side reaction with LPSCl and increase the Coulombic efficiency, H-LNMO particles were coated with nanometer-thick layers of Al_2_O_3_ by atomic layer deposition (ALD) (see the Methods section of the Supporting Information).^40−43^ The XRD pattern of Al_2_O_3_-coated H-LNMO does not contain additional peaks ascribable to Al_2_O_3_, thus suggesting the deposition of a thin and amorphous layer (Figure 3a).^44^ The presence of an Al_2_O_3_ layer was further confirmed by X-ray photoemission spectroscopy (XPS), where peaks characteristic of Al_2_O_3_ were observed in the Al 2p and 2s spectra at 75.9 and 120.8 eV, respectively (Figure 3b).^45,46^ Scanning transmission electron microscopy (STEM) highlighted a discrete change in the atomic array between the crystalline LNMO and an amorphous Al_2_O_3_ layer estimated to be about 1 nm thick after five cycles of ALD (Figure 3c). As the number of ALD cycles increased from two to eight, the thickness of Al_2_O_3_ also increased from 0.4 to 1.4 nm, as confirmed by both STEM and ellipsometry (Figures S6 and S7).

**Figure 3:**
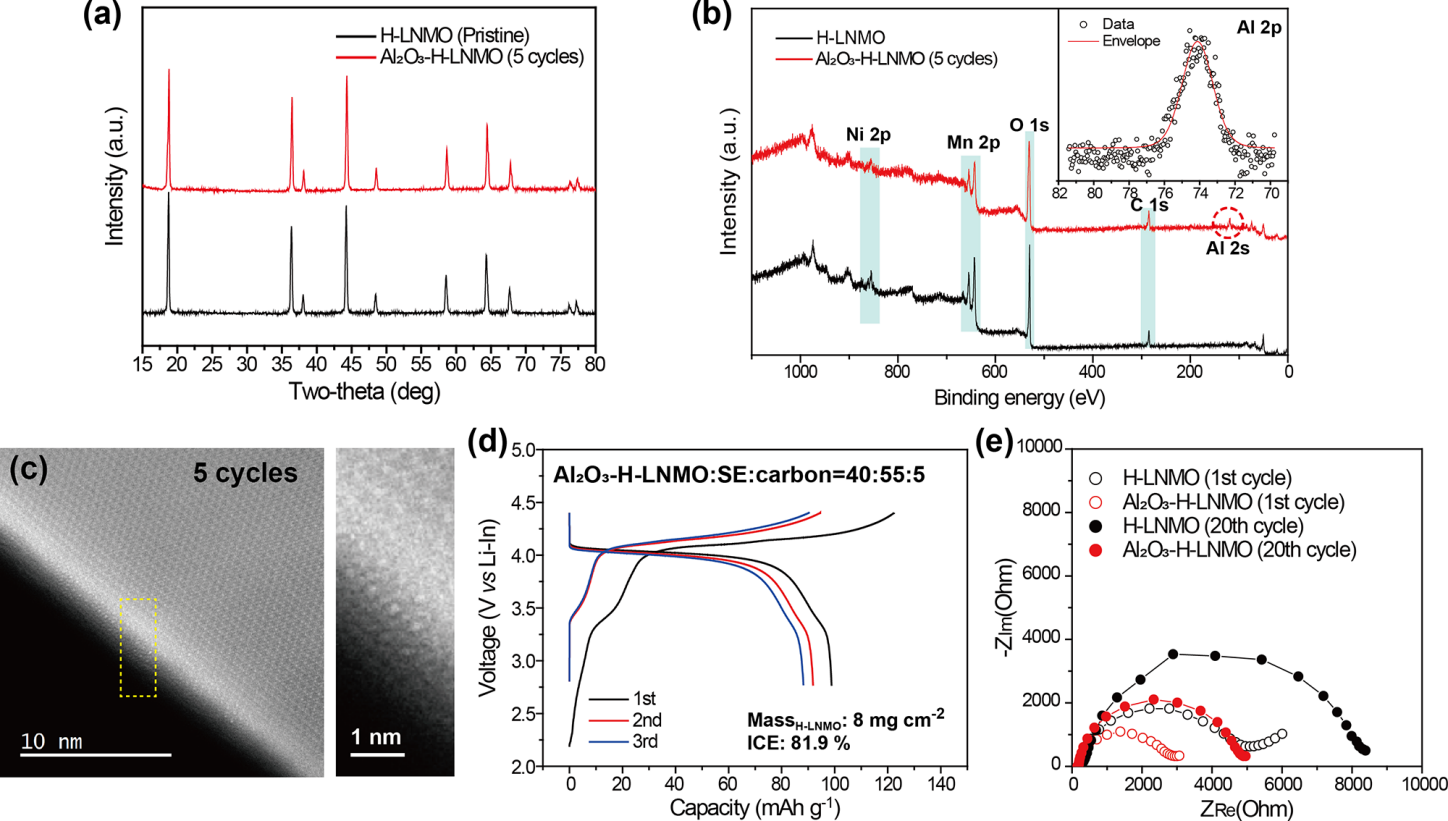
Comparative (a) XRD patterns and (b) XPS survey spectra of H-LNMO and Al_2_O_3_-coated H-LNMO with five cycles of ALD. The inset shows a magnified view of the Al 2p region for the Al_2_O_3_-coated H-LNMO. (c) STEM images of Al_2_O_3_-coated H-LNMO after five ALD cycles. (d) GCD curves of SSBs with Al_2_O_3_-coated H-LNMO after five ALD cycles at a rate of 0.1 C. (e) EIS spectra of SSBs with Al_2_O_3_–H-LNMO and H-LNMO cathode composites after the first and 20th cycles.

To identify the optimal thickness of the Al_2_O_3_ coating layer, H-LNMO samples with Al_2_O_3_ surface layers deposited by a different number of ALD cycles (two, five, and eight cycles) were evaluated electrochemically. As shown in Figure S8a, the discharge capacity of H-LNMO increased from 83.4 to 99.4 mAh g^–1^ with two cycles of ALD. In addition, the initial Coulombic efficiency of H-LNMO with two ALD cycles improved to 81.4%, thus suggesting the mitigation of side reactions between LPSCl and coated LNMO at high operating potentials. Al_2_O_3_-coated H-LNMO with five ALD cycles exhibited an electrochemical performance similar to that of H-LNMO with two ALD cycles (Figure 3d). However, a comparison of the cycling performance between these two samples after 40 cycles revealed that the capacity retention of H-LNMO with fve ALD cycles was 62.1%, significantly higher than that of H-LNMO with two ALD cycles (53.7%) (Figure S8b).

As previously reported in a separate study, a coating layer combined synergistically with a hollow structure can effectively mitigate the volume change of the cathode material, and the constraint becomes increasingly significant as the thickness of the surface layer increases.^18^ Therefore, we speculate that the lower volume change of H-LNMO brought about by 1 nm of ALD-coated Al_2_O_3_ provides for improved capacity retention compared with thinner coating layers. Nonetheless, when the number of ALD cycles increased to eight, the capacity decreased to 91.1 mAh g^–1^, as the insufficient Li-conductivity of the Al_2_O_3_ layer hinders Li-ion migration (Figure S8a). In summary, 1 nm of Al_2_O_3_ deposited using five cycles of ALD provided the best compromise between cycling stability and discharge capacity in this study, and we therefore focused the rest of the investigation on this system (denoted as Al_2_O_3_–H-LNMO). Plasma FIB cross-section SEM images of the PC-LNMO composite cathode exhibited contact loss between PC-LNMO and LPSCl after a few cycles, whereas the Al_2_O_3_–H-LNMO composite cathode maintained intimate interfacial contact between Al_2_O_3_–H-LNMO and LPSCl after 100 cycles, thus confirming that the hollow microstructure coupled with Al_2_O_3_ layer effectively mitigates the volume change of the LNMO cathode (Figures S9 and S10).

XPS measurements of H-LNMO and Al_2_O_3_–H-LNMO composite cathodes were performed to identify the influence of the Al_2_O_3_ coating layer on decomposition processes. The presence of oxygenated sulfur and phosphorus compounds such as sulfites and phosphates, which result from the reaction between LNMO and LPSCl, was confirmed by the XPS analysis of the H-LNMO composite cathode after 50 cycles, whereas these compounds were barely detected in the Al_2_O_3_–H-LNMO composite cathode (Figure S11).^47,48^ This reveals that the Al_2_O_3_ surface layer effectively suppresses the interfacial reaction between LPSCl and H-LNMO, thus leading to the enhanced cycling performance of Al_2_O_3_–H-LNMO.

Electrochemical impedance spectroscopy (EIS) spectra of H-LNMO/LPSCl/Li–In and Al_2_O_3_–H-LNMO/LPSCl/Li–In are shown in Figure 3e. It was not possible to deconvolute the contributions of the cathode and the anode to the reaction resistance, as previously reported.^22^ Therefore, an equivalent circuit model combining the contributions from both the anode and the cathode into one reaction resistance, *R*_*E*_ (where E stands for electrodes) was used to interpret the EIS spectra. The difference in resistance observed between the H-LNMO/LPSCl/Li–In and Al_2_O_3_–H-LNMO/LPSCl/Li–In systems can be directly attributed to the effect of the Al_2_O_3_ layer, as the anode is identical across both samples. The *R*_*E*_ values of the coated and noncoated H-LNMO samples were 0.57 and 0.96 kΩ cm^2^, respectively, after the first discharge (Figure S12 and Table S1). After 20 cycles, the increase in resistance was more significant for the H-LNMO system than for the Al_2_O_3_–H-LNMO system, highlighting the efficacy of the protective layer in terms of stabilizing the cathode–SE interface at high potentials. The effect of the active material fraction in the composite cathode was explored by comparing Al_2_O_3_–H-LNMO/LPSCl/VCF compositions with material ratios of 40:55:5 and 70:25:5. Although high cathode fractions naturally lower the fraction of solid electrolyte and increase the tortuosity of ionic paths, both composite cathodes delivered similar capacities of 94.9  (70 wt %) and 99.4 mAh g^–1^ (40 wt %), which confirms the facile Li-ion diffusion in H-LNMO (Figure S13).

To demonstrate the benefits of H-LNMO in a realistic cathode configuration, a film-type composite cathode was fabricated using a polytetrafluoroethylene (PTFE)-based dry processing method^49^ (Figure S14). The fibrous network produced by PTFE under the shear stress of repeated grinding steps forms a cohesive composite cathode with good ionic and electronic transport.^50,51^ The GCD curves of Al_2_O_3_–H-LNMO/LPSCl/Li–In with pellet- and film-type composite cathodes are displayed in Figure 4a and b, respectively. The cells were cycled under constant current–constant voltage (CC–CV) charge and CC discharge to promote the full delithiation of the H-LNMO lattice. The first discharge capacities were 105.5 and 89.8 mAh g^–1^ for the pellet- and film-type cathodes, respectively, at a C-rate of 0.1 C. After 100 cycles, the capacity retention of the cells cycled with the pellet- and film-type composite cathodes were 62.1% and 70.1%, respectively; thus, both cathodes displayed stable cycling performance even with a high active material loading (14 and 27 mg cm^–2^ for pellet- and film-type composite cathodes, respectively) (Figure 4c). The enhanced capacity retention of the film-type composite cathode can be attributed to the ability of the PTFE fibrils to maintain the contact between LPSCl, Al_2_O_3_–H-LNMO, and VCF upon cycling. The areal capacities of pellet- and film-type composite cathodes were 1.51 and 2.46 mAh cm^–2^, respectively, which were 7–10× higher than those of previously reported SSBs composed of LNMO and a sulfide electrolyte,^22,23,52−56^ thus confirming the importance of microstructure engineering cathode secondary particles to achieve high areal capacities and long-term cyclability in SSBs (Figure 4d and e and Table S2).

**Figure 4:**
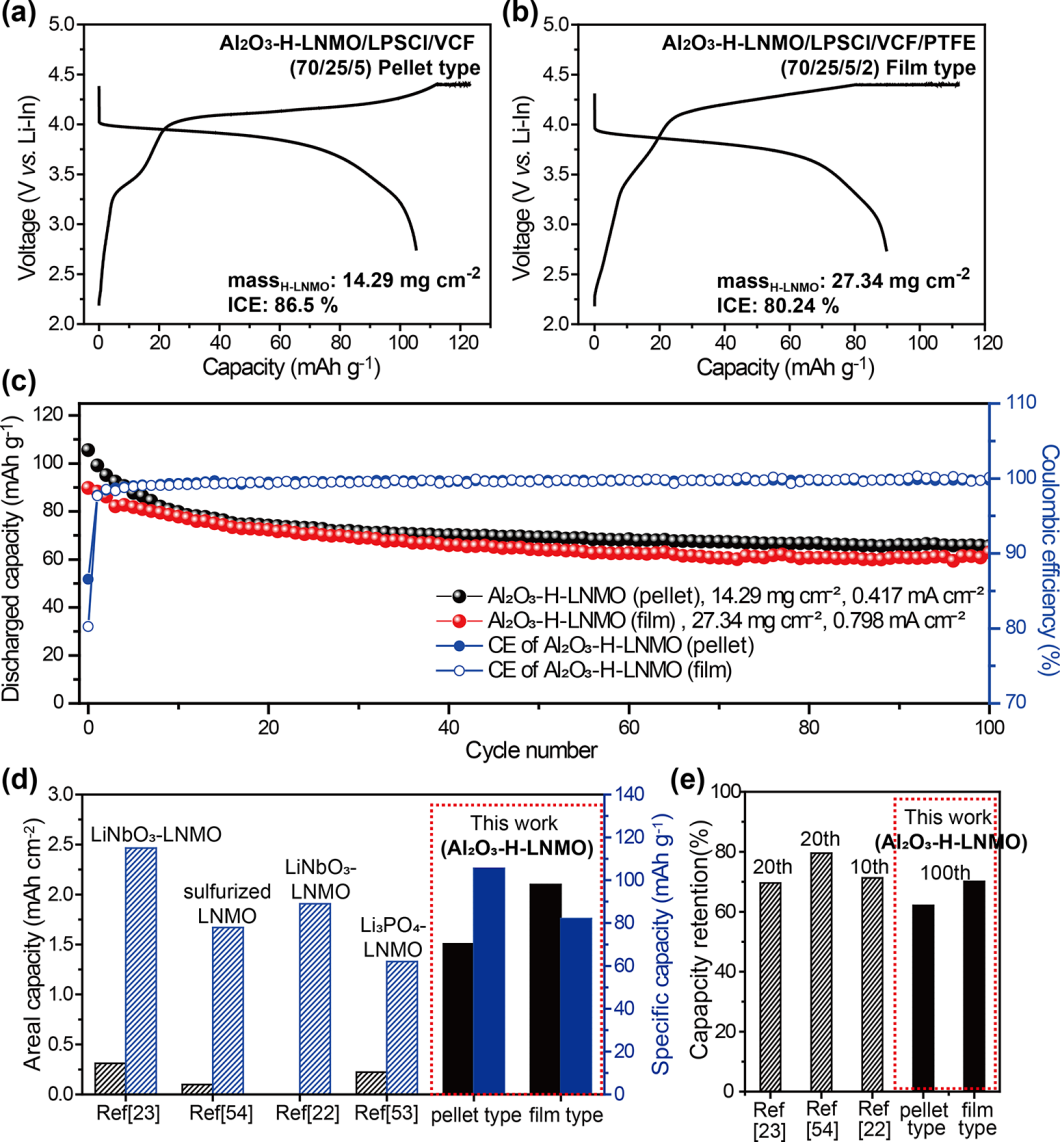
Comparative GCD curves of Al_2_O_3_–H-LNMO/LPSCl/Li–In cells with (a) pellet- and (b) film-type composite cathodes (70:25:5) at a rate of 0.1 C under the CC–CV mode. (c) Cycling performance of SSBs with pellet- and film-type Al_2_O_3_–H-LNMO composite cathodes. The rate was increased to 0.2 C after the first three cycles, which were performed at a rate of 0.1 C. Comparison of (d) the initial areal and specific capacities and (e) the capacity retention of SSBs composed of LNMO and solid sulfide electrolytes between this study and reference data.

In conclusion, we have demonstrated that a hollow microstructure coupled with a stable surface layer significantly improves both the cycling performance and the rate capability of LNMO-based cathodes with a high active material loading. The hollow microstructure reduces the Li-ion diffusion path, leading to lower overpotentials and faster reaction kinetics at the electrode surface. The hollow secondary particle morphology coupled with the Al_2_O_3_ surface coating effectively mitigates both the volume change and the induced stress level in LNMO during lithium insertion and extraction, minimizing contact loss between the cathode and the solid electrolyte and resulting in improved cycling stability. This study highlights the importance of cathode microstructure engineering in SSBs and provides design strategies that can be extended to more traditional cathode chemistries.

## Supporting Information Available

Details on materials and methods, PSD curves of H-LNMO and PC-LNMO, EIS spectra and Arrhenius plot of LPSCl, SEM images of PC-LNMO, STEM images of Al_2_O_3_-coated H-LNMO after ALD cycles, and GCD curves of H-LNMO with an Al_2_O_3_ layer formed under a different number of ALD cycles (PDF)
