# Supplementary material for: LiNi0.5Mn1.5O4 Cathode Microstructure for All-Solid-State Batteries
Source: Nano Lett. 2022 Sep 7;22(18):7477–83. doi: 10.1021/acs.nanolett.2c02426 (PMC9523706; doi:10.1021/acs.nanolett.2c02426)
Supplement: Supplementary file 1 — nl2c02426_si_001.pdf [file nl2c02426_si_001.pdf]

Supporting Information

$LiNi_{0.5}Mn_{1.5}O_4$ cathode microstructure for all-solid-state batteries

Hyeon Jeong Lee[a, b, c †], Xiaoxiao Liu[a, d, †], Yvonne Chart[a, b], Peng Tang[a], Jin-Gyu Bae[e], Sudarshan Narayanan[a, b], Ji Hoon Lee[e], Richard J. Potter[f], Yongming Sun[d], and Mauro Pasta[a,b,*]

[a]Department of Materials, University of Oxford, Parks Road, Oxford OX1 3PH, United Kingdom

[b]The Faraday Institution, Harwell Campus, Quad One, Becquerel Avenue, Didcot OX11 0RA, United Kingdom

[c]Division of Chemical Engineering and Bioengineering, Kangwon National University, Chuncheon, 24341, Republic of Korea

[d]Wuhan National Laboratory for Optoelectronics, Huazhong University of Science and Technology, Luoyu Road 1037, Wuhan 430074, China

[e]School of Materials Science and Engineering, Kyungpook National University, Daegu 41566, Republic of Korea

[f]Department of Mechanical, Materials and Aerospace Engineering, University of Liverpool, Brownlow Street, Liverpool L69 3GH, United Kingdom

[†]H. J. Lee and X. Liu contributed equally to this work

[*]Corresponding author: mauro.pasta@materials.ox.ac.uk

Methods

Synthesis of Hollow-$LiNi_{0.5}Mn_{1.5}O_4$(H-LNMO)

All reagents were purchased from Sigma-Aldrich and used without further purification. To obtain the $Ni_{0.25}Mn_{0.75}CO_3$ precursor of H-LNMO, 1 mmol of nickel(II) acetate tetrahydrate ($Ni(OCOCH_3)_2{\cdot}4H_2O$) and 3 mmol of manganese(II) sulfate monohydrate ($MnSO_4{\cdot}H_2O$) were first dissolved in a mixture of 12.5 mL of deionized (DI) water and 6.25 mL of ethanol. After vigorous stirring, 6.5 mL of 0.5 M ammonium bicarbonate (NH_4HCO_3) and 6 mL of 0.5 M sodium carbonate (Na_2CO_3) were added to the solution followed by additional stirring for 6 h at 25 °C. The resulting suspension was transferred to a 100 mL Teflon-lined autoclave reactor, which was sealed and heated at 140 °C for 10 h. After the reaction was completed, the $Ni_{0.25}Mn_{0.75}CO_3$ precursor was rinsed with distilled water and ethanol several times and dried at 60 °C under vacuum for 10 h. Finally, the obtained $Ni_{0.25}Mn_{0.75}CO_3$ was mixed with a stoichiometric amount of lithium carbonate (Li_2CO_3) and heated for 10 h at 900 °C in air followed by additional heating at 700 °C for 4 h. Polycrystalline-LNMO (PC-LNMO)and single crystal LNMO (SC-LNMO) were purchased from MTI and used as control samples.

Al_2O_3 atomic layer deposition (ALD)

The powder samples were loaded into a custom-built fluidised bed powder adapter under argon using a purge glovebox before being rapidly transferred in air to the ALD reactor. Stainless steel filters (SSU25 with a porosity of ∼37% and average pore size of 27 µm, AmesPore) above and below the powder were used in the adapter to enable gases to pass through the cell while confining the powder. Al_2O_3 was deposited by thermal ALD using trimethyl-aluminium (TMA, SAFC Hitech) and distilled water vapour in an Oxford Instruments OpAL reactor. Precursors were held at room temperature and delivered via vapour draw. The reactor was modified to accommodate the powder adapter and a process-controlled 'hold' valve was added to the pump line to enable extended precursor exposures. Coatings were carried out at a reactor temperature of 150°C. A soft pump-down step was used to evacuate the reactor to prevent violent degassing of the powder bed. Powders were degassed for 15 min before coating using pump/purge cycles. Films were deposited using the following sequence (where N is the number of ALD cycles) with 400 sccm or argon flow during purge steps (Figure S15). At the end of the process, the adapter was removed from the ALD reactor and transferred back to the purge glovebox for powder decanting into glass ampules. The growth rate of the Al_2O_3 was estimated using a Rudolph Auto EL IV ellipsometer operating at 633 nm using silicon 'witness' samples placed outside of the adapter on the main reactor heated platen. The thickness of Al_2O_3 was found to increase in a linear fashion with increasing ALD cycles (measurements were taken for 2, 5, 8 and 50 cycles) and the slope of the linear fit gave a nominal growth rate of ∼0.15 nm/cycle.

Characterisation

X-ray diffractometry (XRD, Rigaku Miniflex) was carried out with a Cu Kα radiation source in a nitrogen-filled glovebox with O_2 and H_2O contents less than 1 ppm. The particle size distribution of H-LNMO was measured using dynamic light scattering (DLS) with a Partica LA-960V2 analyser (HORIBA). Scanning transmission electron microscopy (STEM, JEOL ARM-200F) micrographs, collected at an acceleration voltage of 200 kV, were used to estimate the thickness of the Al_2O_3 layer. The Al_2O_3-H-LNMO was dispersed in ethanol and sonicated for 10 min. The supernatant was dropped on a lacey carbon film on a Cu TEM grid. The TEM grids were dried under vacuum at 60 °C for 10 h. Field emission-scanning electron microscopy (FE-SEM, Merlin) was used to observe the microstructure and morphology of the H-LNMO samples and composite cathode. For all ex-situ experiments, the samples were transferred using vacuum transfer vessels to avoid air exposure. XPS spectra were collected using a Phi XPS VersaProbe III with an Al Kα X-ray source. Data quantification was performed using CasaXPS software. All spectra were charge-referenced to the disordered C1s component at a binding energy of 284.8 eV.

Ex situ XANES analyses

Ex situ XANES measurement was conducted at the 7D beamline at Pohang Accelerator Laboratory (PAL) in the Republic of Korea. The cathode composites with H-LNMO and PC-LNMO were prepared by electrochemical charging (3.4 V, 4.0 V and 4.4 V vs Li-In) and discharging processes (2.75 V vs Li-In) at a rate of 0.1C. The composite cathodes were separated from the SSBs and sealed in Kapton tape to prevent air exposure during the XANES measurement. The sample was prepared in an Ar-filled glovebox. Ni K-edge spectra were obtained in fluorescence mode at room temperature. Calibration for each spectrum was performed by recording the Ni metal foil simultaneously. The storage ring energy was set to 3 GeV under top-up mode with a maximum current of 300 mA.

Electrochemical measurement

To prepare the composite cathode, $LiNi_{0.5}Mn_{1.5}O_4$ particles, Li_6PS_5Cl (LPSCl, Ampcera) and vapour-grown carbon nanofibers (VCF, Sigma-Aldrich) with two different mass ratios of 40:55:5 and 70:25:5 were mixed by a vibratory ball mill with gradually increasing speed from 1200 to 1800 rpm for 15 mins. The Li-In foil anode (atomic ratio: 25% Li and 75% In) was prepared by melting indium beads and lithium metal foil at 600°C followed by a cold rolling process and the final thickness of the Li-In anode was 100 µm. SSBs were assembled using a polyether ether ketone (PEEK) mould and two stainless steel plungers with a diameter of 5 mm. The Li-ion conductivity of the LPSCl solid electrolyte is 0.68 mS cm^{-1} at 30 °C and its activation energy is 0.29 eV (Figure S16 and Table S3). For the pellet-type composite cathode, 4 mg of the composite was spread on the pre-pressed 20 mg LPSCl electrolytes. The film type cathode composite was prepared by the following steps. First, 4 mg of PTFE were added to 200 mg of the composite cathode, ground at 80°C for 20 min, and then pressed by repeated rolling. Subsequently, the resultant film was cut into 5 mm diameter disks using a hollow punch, and the disk-type electrode was placed on the pre-pressed 20 mg LPSCl electrolytes. The total mass of the disk-type electrode was 39~40 mg cm^{-2}. Then the stacked layers were further pressed into a dense pellet by compression in the PEEK mould at 500 MPa for 5 mins. A Li-In disk was placed on the opposite side of the electrolyte as an anode. Stainless-steel rods were used as current collectors. A pressure of 50 MPa was applied during cell cycling. All the cells were fabricated in an Ar-filled glovebox (O_2 and H_2O levels <1 ppm) to avoid reactions with moisture. The electrochemical tests were carried out by galvanostatic charge-discharge measurements in the potential range of 2.75–4.4 V (vs Li^+/Li–In) using a battery cycler (VMP3, Biologic, France) at the temperatures of 30°C. For high mass loading composite cathodes, constant current-constant voltage (CC-CV) mode was used for charge process to reduce the battery polarisation. The C-rate is defined based on 1C = 146 $mAhg^{-1}$. Electrochemical impedance spectroscopy (EIS) measurements were performed using a frequency response analyser (VMP3, Biologic) over the frequency range of 0.01 Hz–1 MHz with a voltage amplitude of 10 mV.

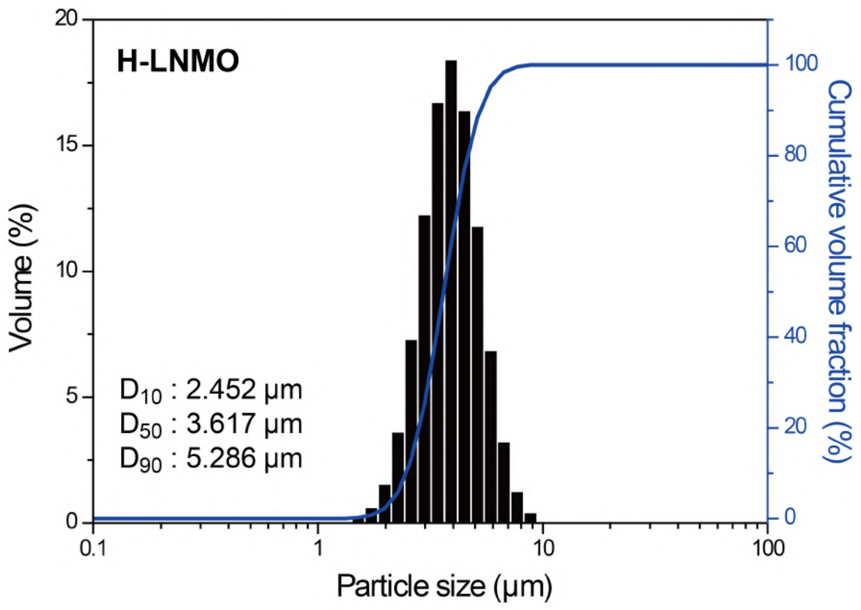

Figure S1. Particle size distribution of as-synthesized H-LNMO.

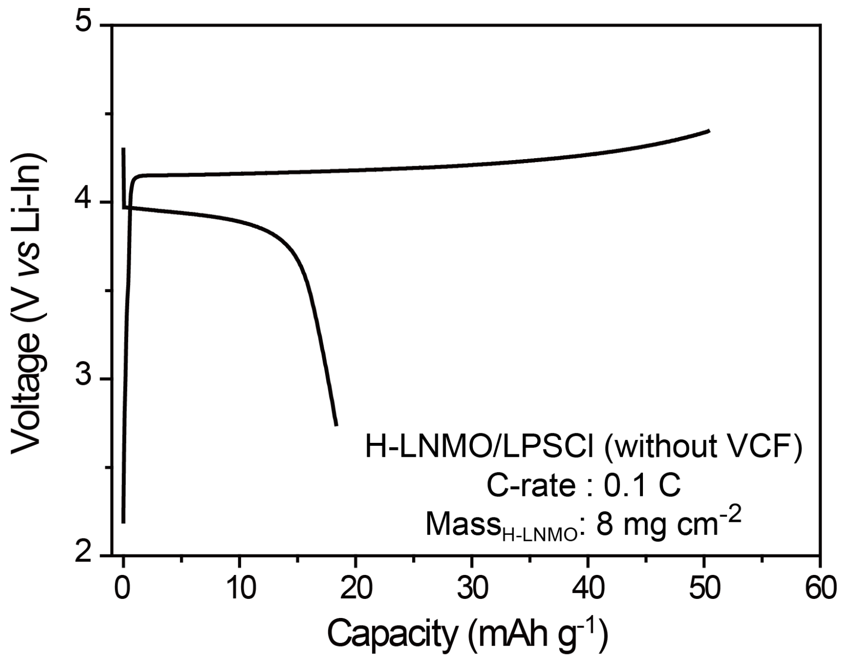

Figure S2. GCD curve of SSB with H-LNMO composite cathode without VCF at a C-rate of 0.1C. The overpotential of the H-LNMO/LPSCl composite cathode increased and the discharge capacity sharply decreased to 20 mAh g^{-1}, confirming that the carbon additives are indispensable to enable homogeneous current distribution in the cathode layer.

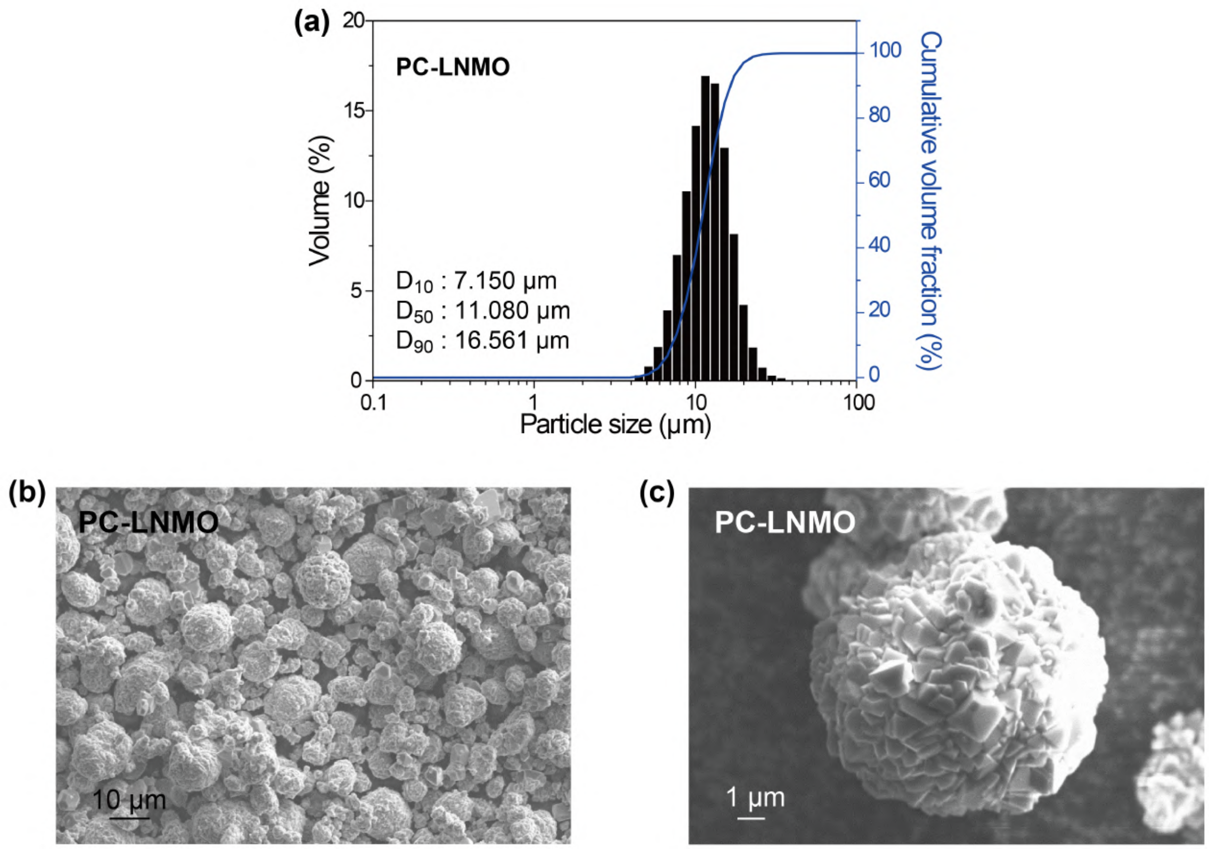

Figure S3. (a) Particle size distribution of PC-LNMO. SEM images of PC-LNMO at (b) low and (c) high magnifications.

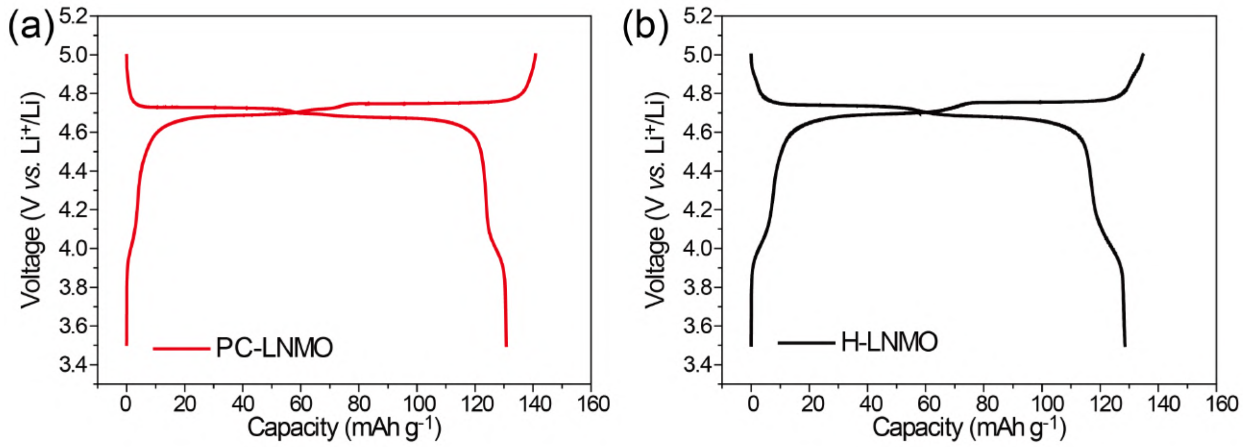

Figure S4. GCD curves of (a)PC-LNMO and (b) H-LNMO in LP30 electrolyte at a current of 0.1 C in the potential range 3.5–5.0 V (vs Li^+/Li).

H-LNMO and PC-LNMO were cycled in commercial LP30 liquid electrolyte (1 M $LiPF_6$ in EC/DMC (1:1, v:v)): discharge capacities of 128.5 and 130 mAh g^{-1}, respectively were observed at a C-rate of 0.1C.

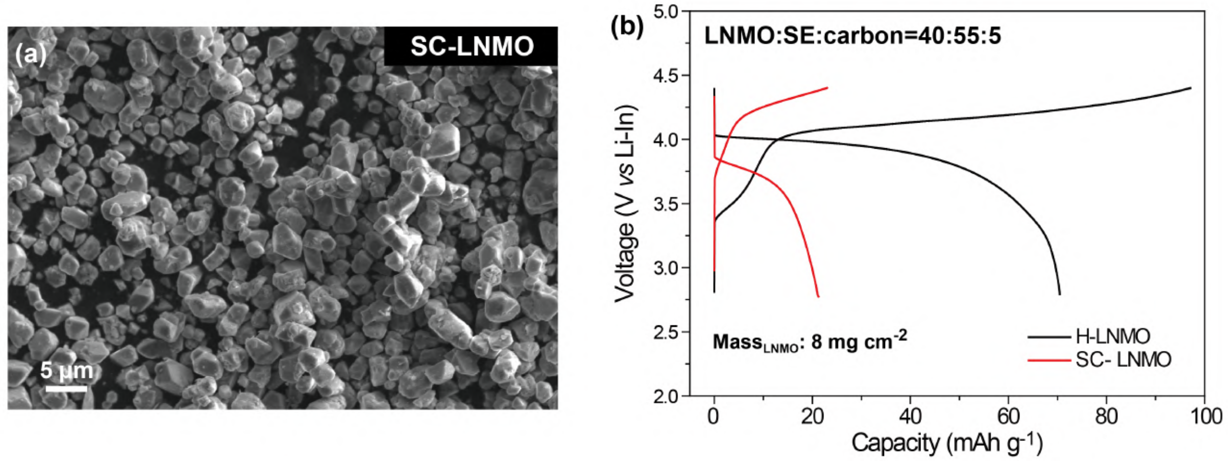

Figure S5. (a) SEM images of single crystal LNMO (SC-LNMO). (b) Comparative GCD curves of SSBs with H-LNMO and SC-LNMO composite cathodes at a C-rate of 0.1C.

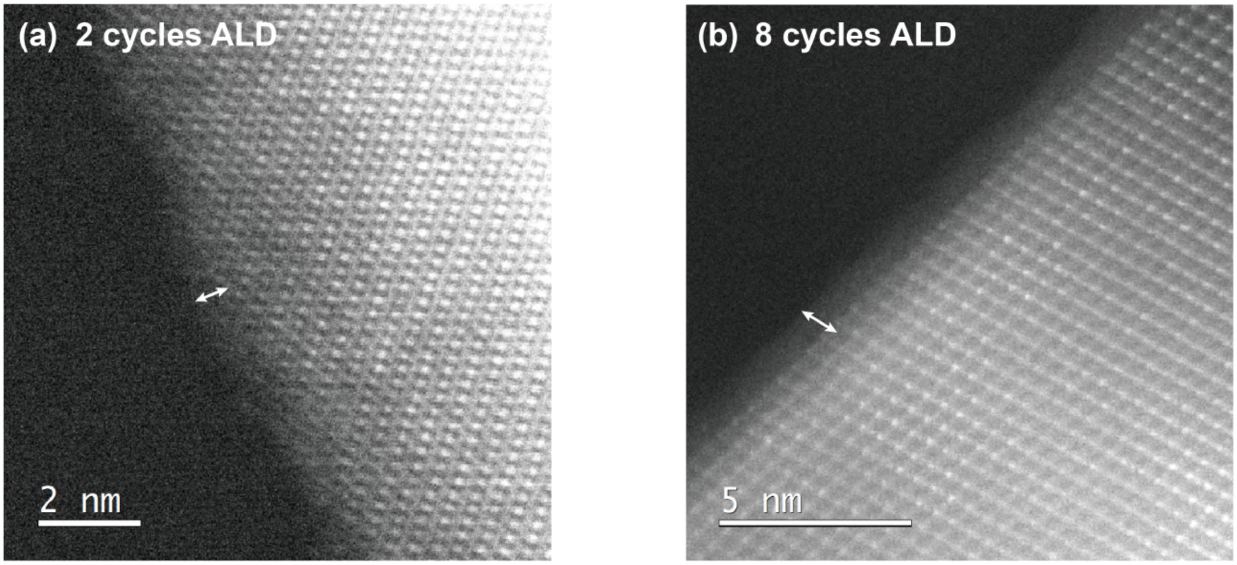

Figure S6. STEM images of Al_2O_3-coated H-LNMO with (a)2 and (b)8 cycles of ALD.

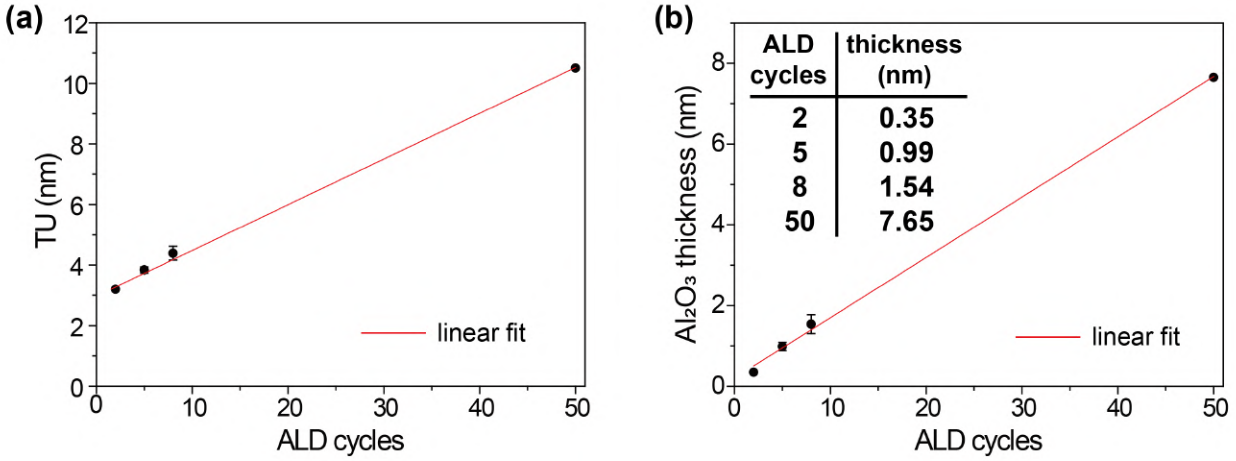

Figure S7. (a) Calculated thickness (TU) of Al_2O_3 film deposited on silicon substrates. (b) Al_2O_3 film thickness as function of the number of ALD cycles. Al_2O_3 film thickness was calculated by subtracting the offset value from each TU value. The offset value(2.85 nm) was obtained from the extrapolated x-intercept of (a).

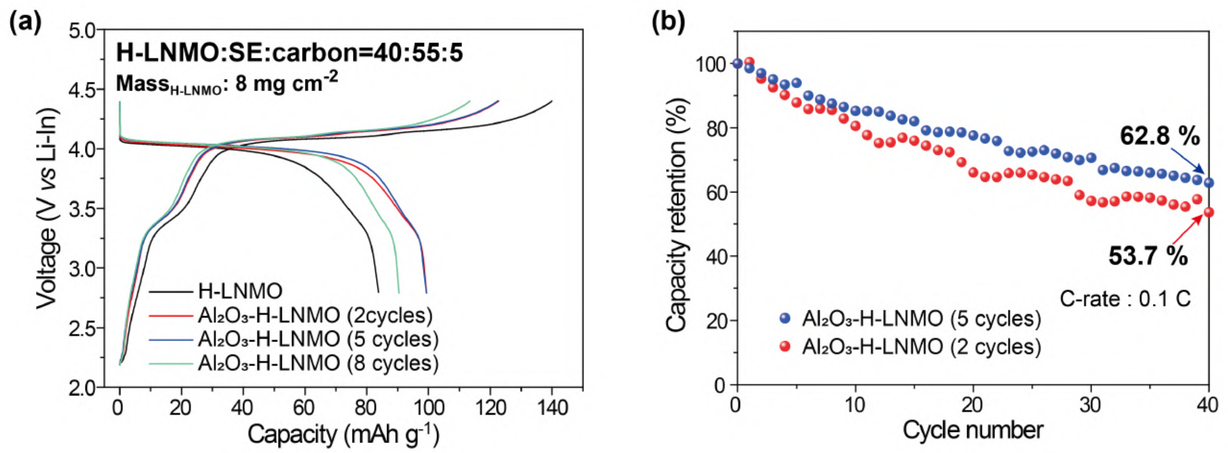

Figure S8. Comparative GCD curves of SSBs with H-LNMOs after 2, 5, and 8 cycles of ALD. (b) Cycling performance of SSBs with H-LNMOs after 2 and 5 cycles of ALD at a c-rate of 0.1 C.

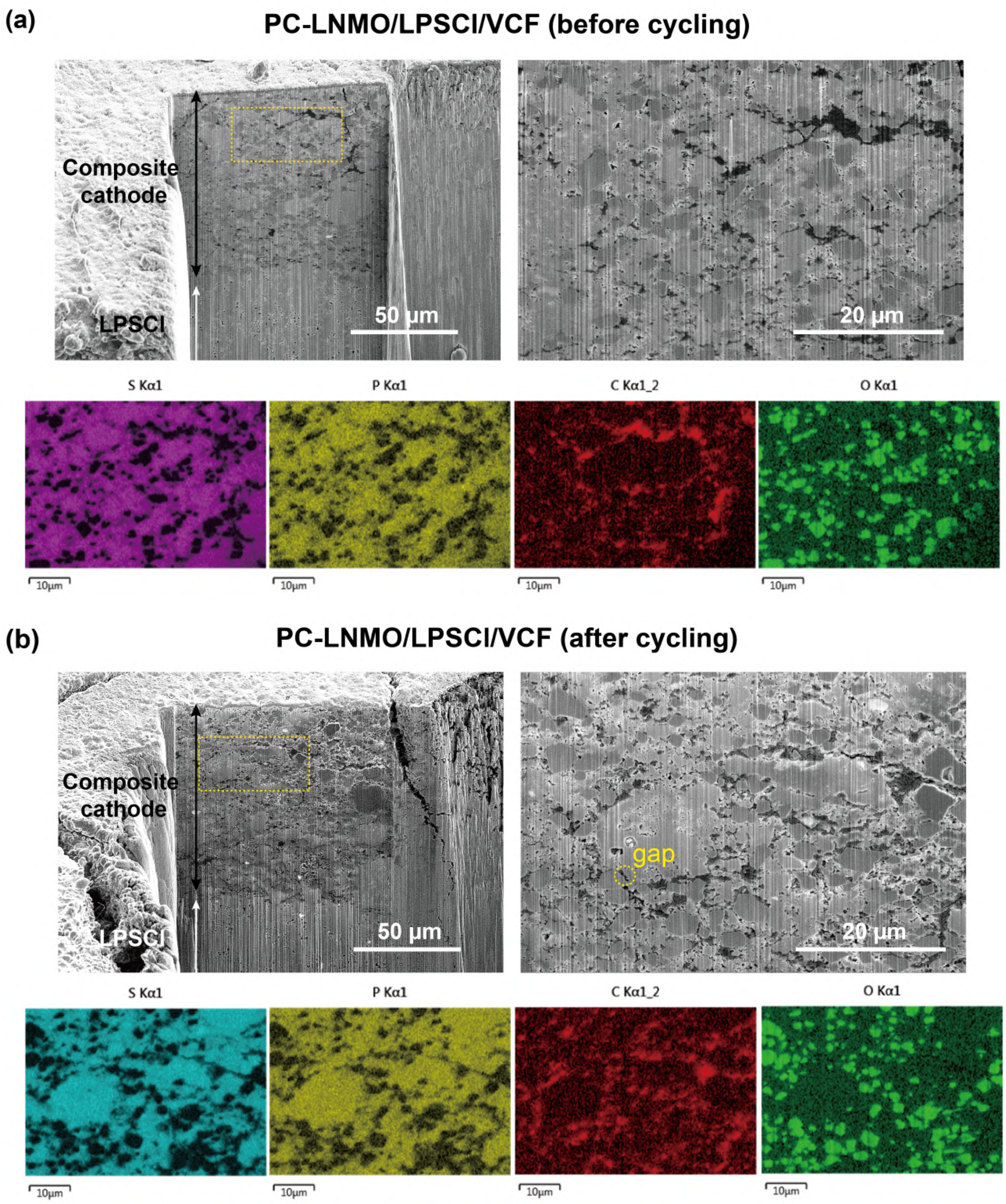

Figure S9. Plasma focused ion beam (PFIB) cross-section SEM images and EDS elemental mappings of PC-LNMO/LPSCl/VCF composite cathodes (a)before and (b)after cycling.

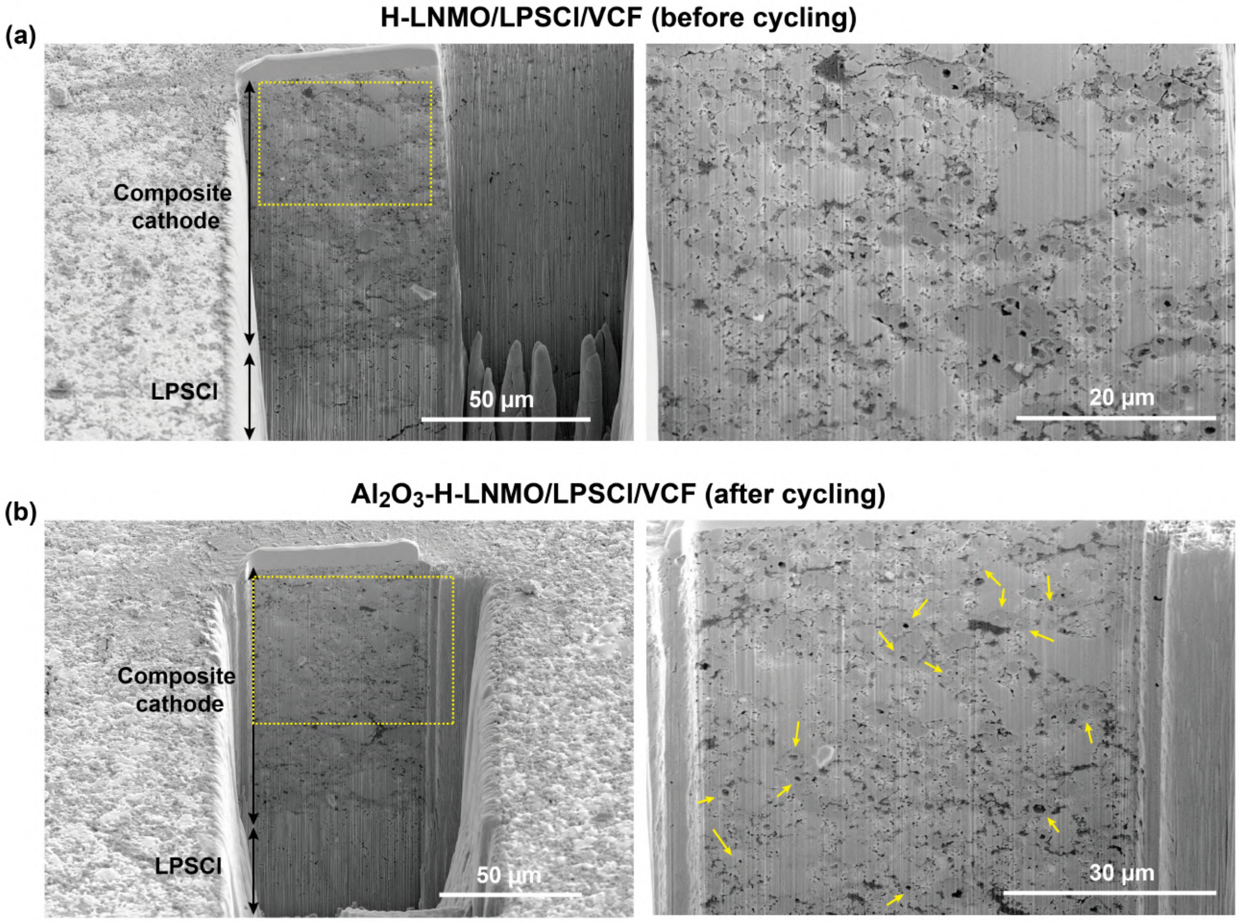

Figure S10. PFIB cross-section SEM images of H-LNMO/LPSCl/VCF composite cathode before cycling and Al_2O_3-H-LNMO/LPSCl/VCF composite cathode after 100 cycles. The yellow arrows in the SEM image of the Al_2O_3-H-LNMO/LPSCl/VCF composite cathode show intimate contact between Al_2O_3-H-LNMO and LPSCl solid electrolyte after cycling.

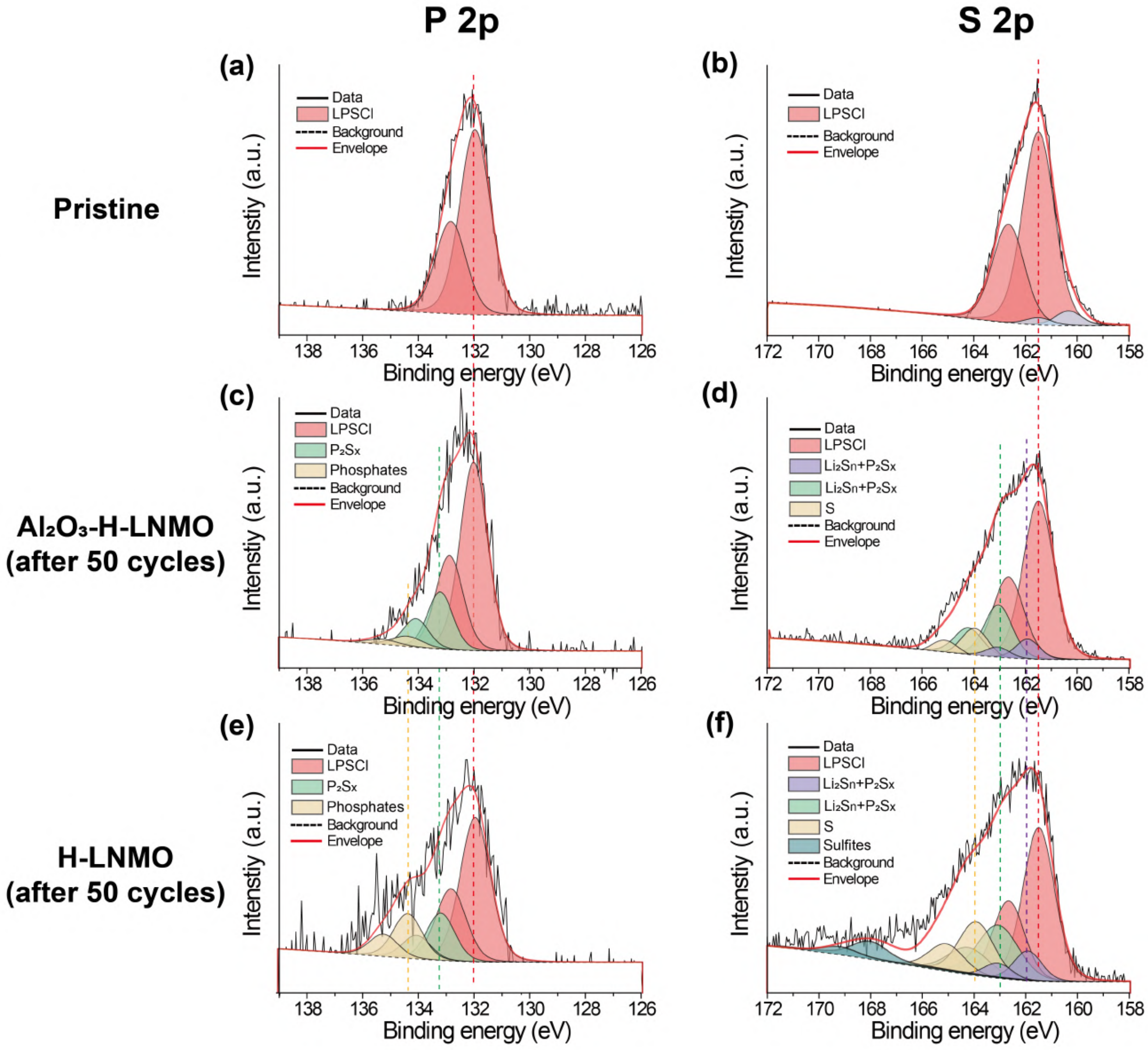

Figure S11. XPS spectra of pristine LPSCl solid electrolyte at P 2p (a) and S 2p (b). XPS spectra of Al_2O_3-H-LNMO and H-LNMO after 50 cycles at the binding energy regions of P 2p (c,e) and S 2p (d,f).

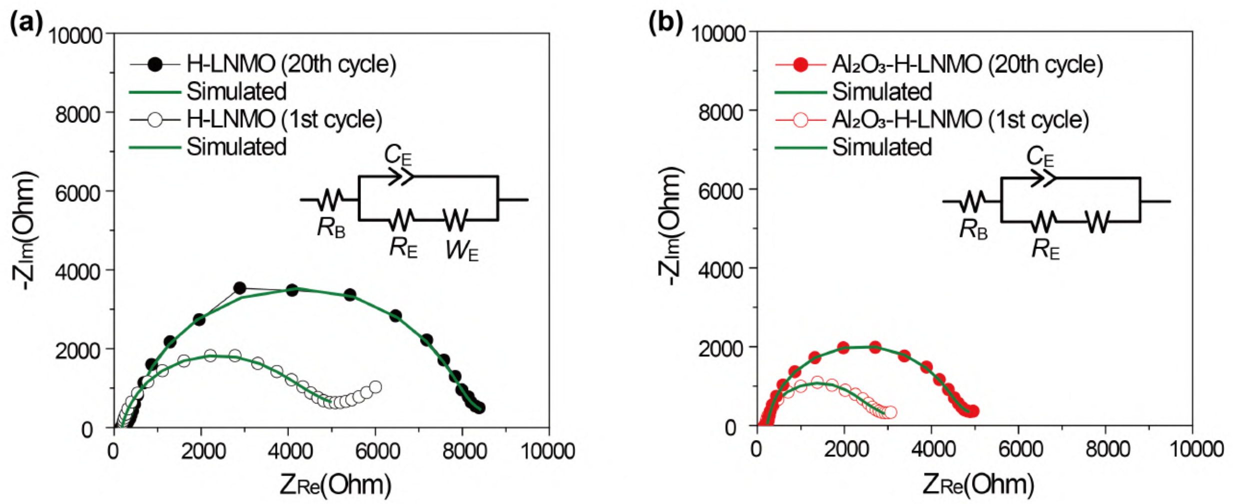

Figure S12. Equivalent circuit models and fitted curves for (a) H-LNMO/LPSCl/Li-In and (b) Al_2O_3-H-LNMO/LPSCl/Li-In SSBs.

**cell**	**cycle**	R_B (Ω)	R_E(**kΩ**)	C_E(**F**)
H-LNMO/LPSCl/Li-In	1st	177.7	4.902	11.09e-6
H-LNMO/LPSCl/Li-In	20th	280.6	8.107	11.09e-6
Al_2O_3-H-LNMO/LPSCl/Li-In	1st	186.6	2.946	11.09e-6
Al_2O_3-H-LNMO/LPSCl/Li-In	20th	230	4.892	11.09e-6

Table S1. Fitting results for the EIS spectra of SSBs with H-LNMO and Al_2O_3-H-LNMO cathode composites after the 1st and 20th cycle.

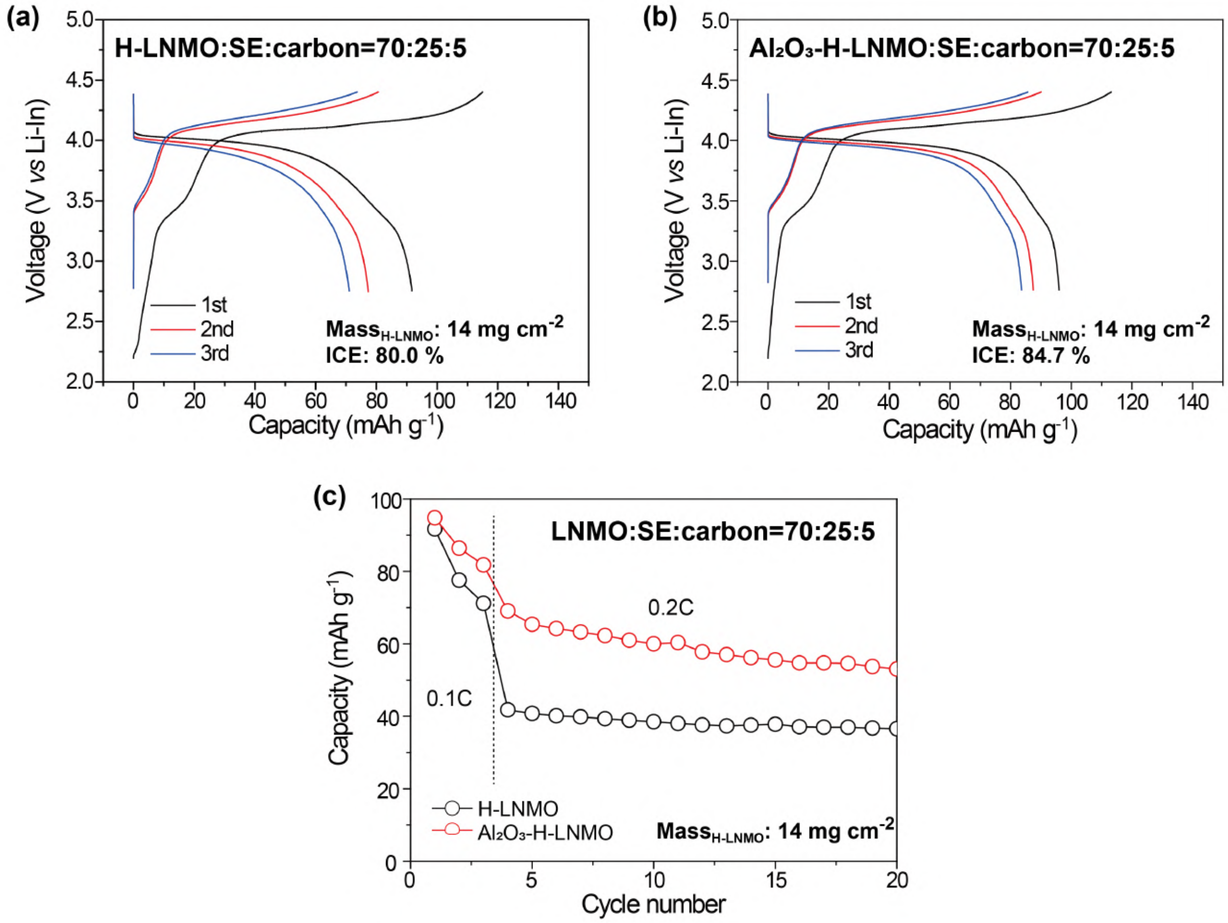

Figure S13. GCD curves of SSBs with (a)H-LNMO and (b)Al_2O_3-H-LNMO composite cathodes with a weight ratio of 70:25:5 (LNMO:LPSCl: VCF, LNMO mass loading of 14 mg cm^{-2}). (c) Comparative cycling performance of H-LNMO and Al_2O_3-H-LNMO composite cathodes.

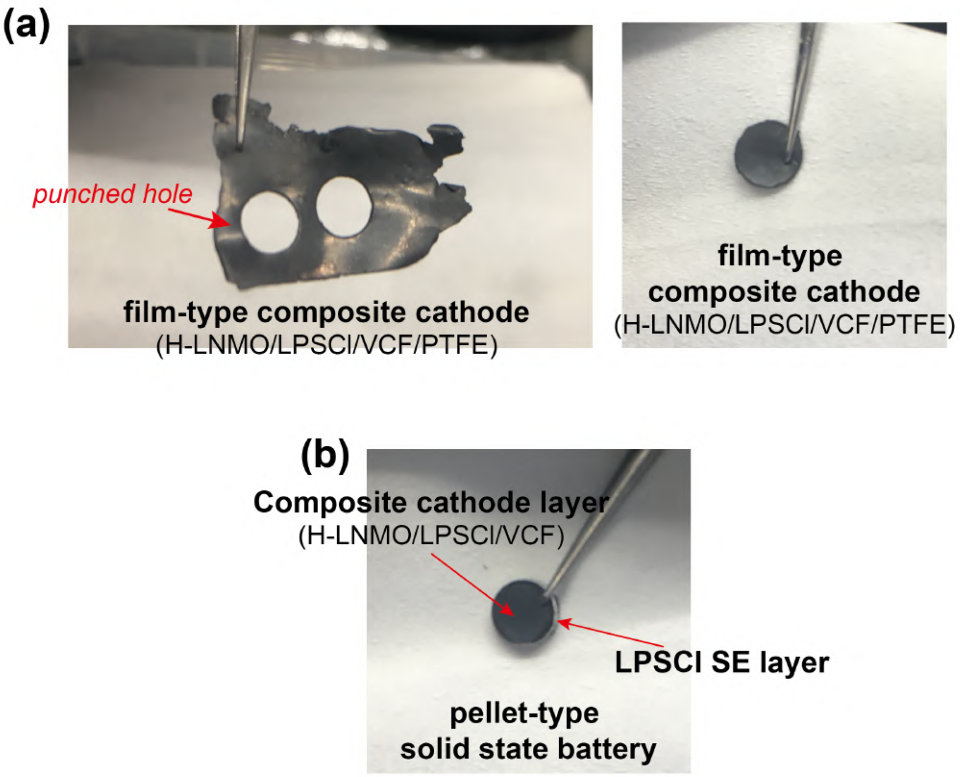

Figure S14. The digital photos of (a) free-standing film-type composite cathode and (b) pellet-type solid-state battery with a size of 5 mm in diameter.

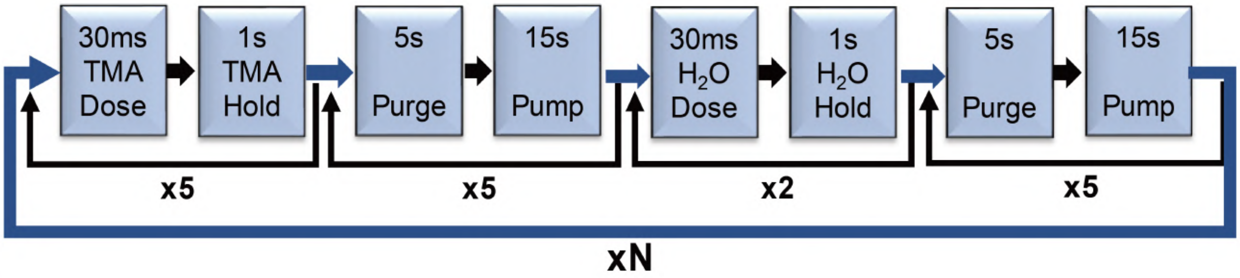

Figure S15. Schematic illustration of the ALD process.

Cathode	mass ratio (LNMO:SE:carbon)	mass loading ($mg_{LNMO}cm^{-2}$)	Solid electrolyte	Specific capacity (mAh g^{-1}) /Current density	Areal capacity (mAh cm^{-2})	Anode	Ref
LNMO thin film	100:0:0 (thin film)	0.45	LiPON	122/ 0.1C	0.0549	Li	[52]
$LiNbO_3$-coated LNMO	38.5:57.5:4	N/A	$Li_{10}GeP_2S_{12}$	80/ 0.05C	N/A	Li-In	[22]
Li_3PO_4-coated LNMO	30:70:6	3.82	Li_2S-P_2S_5	62/0.1C	0.237	In	[53]
$LiNbO_3$-coated LNMO	70:30:0	2.68	LPSCl	115/0.1C	0.308	Li	[23]
Li_3PO_4-coated LNMO	70:30:0	2.68	LPSCl	49.9/0.1C	0.134	Li	[23]
$Li_4Ti_5O_{12}$-coated LNMO	70:30:0	2.68	LPSCl	7/0.1C	0.019	Li	[23]
LNMO thin film	100:0:0 (thin film)	N/A	LiPON	120/0.5C	N/A	Li	[55]
Sulfurized LNMO	50:45:5	1.27	LPSCl	77.9 /0.1C	0.099	$Li_4Ti_5O_{12}$	[54]
$LiNbO_3$-coated LNMO	66:31:3		Li_3YCl_6	91.0 / 20 mA g^{-1}	1.89	Li-In	[56]
Al_2O_3-H-LNMO (pellet type)	70:25:5	14.29	LPSCl	105.5/0.1C<br>92.4/0.2C	1.508 (at 0.209 mA cm^{-2})<br>1.320 (0.417 mA cm^{-2})	Li-In	This study
Al_2O_3-H-LNMO (film type)	70:25:5:2 (LNMO:SE:carbon:PTFE)	27.34	LPSCl	89.8 /0.1C<br>82.1/0.2C	2.455 (at 0.399 mA cm^{-2})<br>2.244 (at 0.798 mA cm^{-2})	Li-In	This study

Table S2. Comparison of electrochemical performance of SSBs based on LNMO and sulfide/halide solid electrolytes.

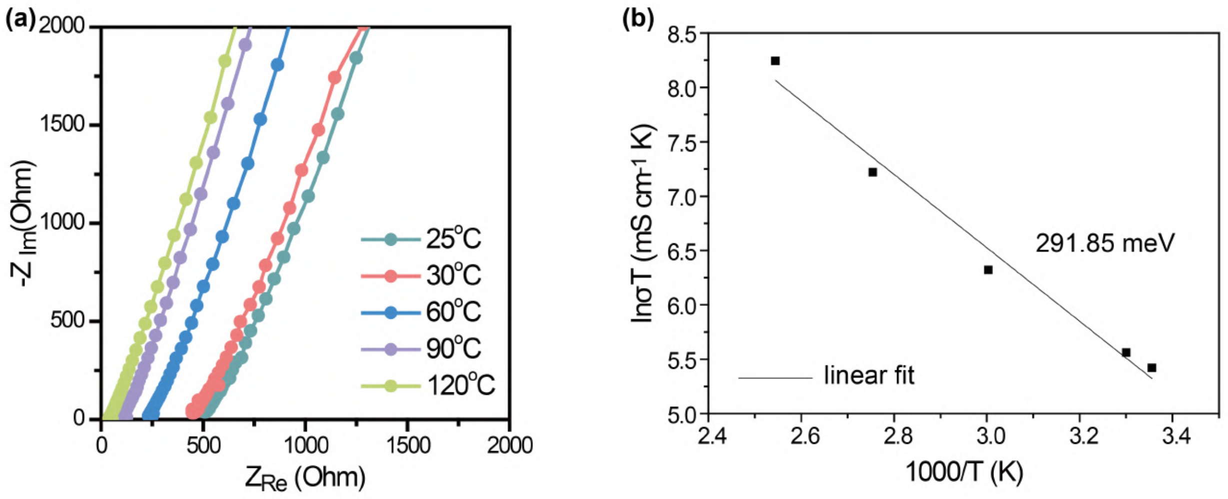

Figure S16. (a) EIS spectra of LPSCl solid electrolyte measrued at 25, 30, 60, 90 and 120 °C. Stainless steel rod was used as blocking electrode. (b) Arrehnius plot of LPSCl solid electrolyte obtained from EIS spectra.

Temperature (°C)	25	30	60	90	120
Conductivity (mS cm^{-1})	0.76	0.86	1.7	3.8	9.7

Table S3. Conductivities of LPSCl solid electrolyte measured at 25-120 °C.
